# Supplementary material for: Identification of photoperiod-induced specific miRNAs in the adrenal glands of Sunite sheep (Ovis aries)
Source: Front Vet Sci. 2022 Jul 22;9:888207. doi: 10.3389/fvets.2022.888207 (PMC9354845; doi:10.3389/fvets.2022.888207)
Supplement: Supplementary file 1 [file Data_Sheet_1.ZIP › Supplementary file/miRDeep2 file.zip!/miRDeep2 file/Know_Structure/SPLP42A2/oar-mir-127.pdf]

miRBase precursor : oar-mir-127
Total read count : 122748
oar-miR-127 read count : 120826
remaining reads : 1922

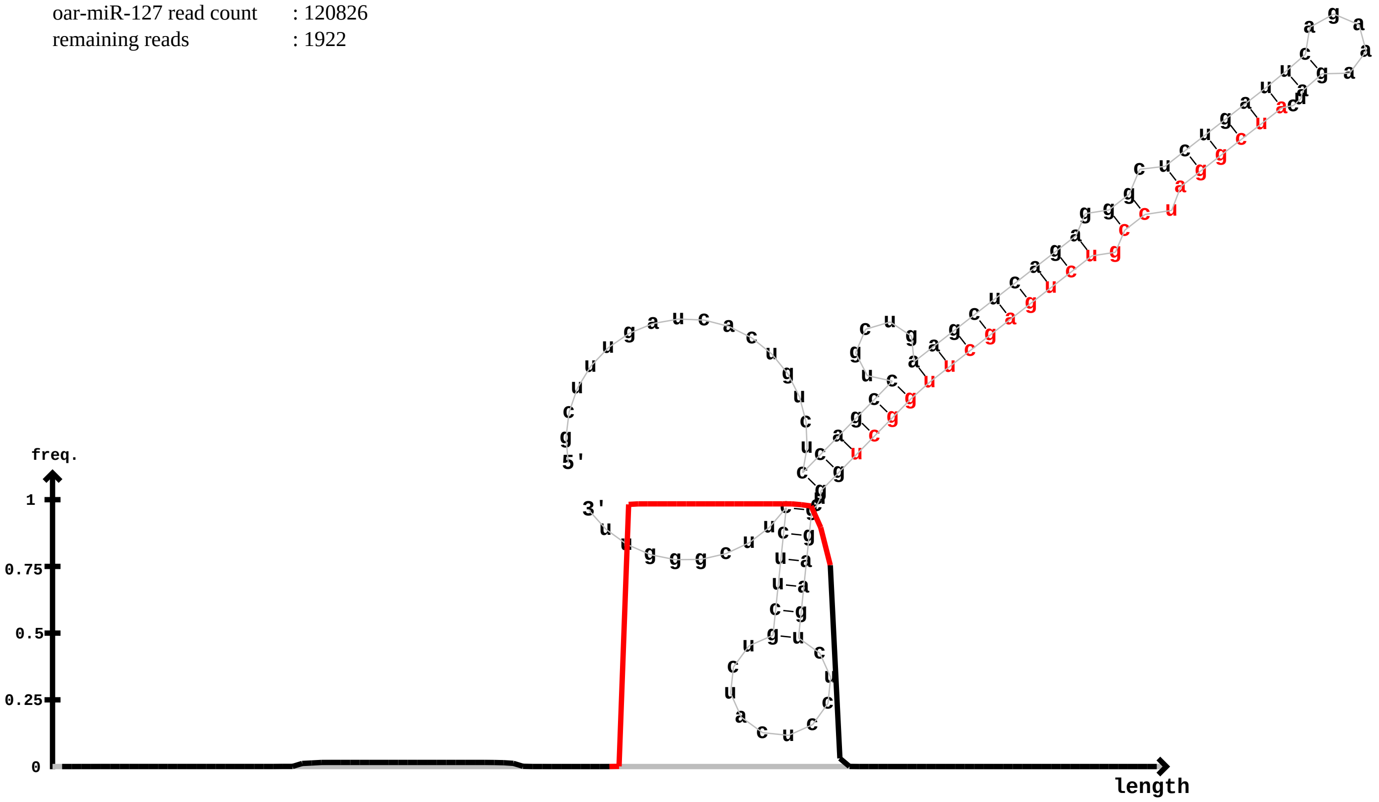


```
                                                          oar-miR-127
5'- gcuuugaucacugucuccagccugcugaagcucagagggcucugauucagaaagaucaucggauccgucugagcuuggcuggucggaagucuccucaucugcuuccuucggguu   -3'   exp
    ................((((((.....(((((((((.((.((((((((.....))..)))))).)).)))))))))))))))..((((((..........))))))........     reads   mm   sample
    gcuuugaucacugucuccagccug..........................................................................................     1       0    seq
    .cuuugaucacugucuccagccu...........................................................................................     1       0    seq
    .cuuugaucacugucuccagccug..........................................................................................     6       0    seq
    ..uuugaucacugucuccagcc............................................................................................     1       0    seq
    ..uuugaucacugucuccagccug..........................................................................................     7       0    seq
    ...uugaucacugucuccagccug..........................................................................................     5       0    seq
    ....ugaCcacugucuccagccug..........................................................................................     1       1    seq
    ....ugaucacugucuccagccug..........................................................................................     2       0    seq
    ......aucacugucuccagccug..........................................................................................     1       0    seq
    ......................ugcugaagcucagagggcucu.......................................................................     5       0    seq
    ......................ugcugaagcucagagggcucug......................................................................     13      0    seq
    ......................ugcugaagcucagagggcucuga.....................................................................     13      0    seq
    ......................ugcugaagcucagagggcucugau....................................................................     1       0    seq
    ......................ugcugaagcucagagggcucugauu...................................................................     4       0    seq
    .......................gcugaagcucagagggcucu.......................................................................     1       0    seq
    .......................gcugaagcucagagggcucuga.....................................................................     2       0    seq
    .......................gcugaagcucagagggcucugau....................................................................     3       0    seq
    ........................cugaagcucagagggcuc........................................................................     1       0    seq
    ........................cuCaagcucagagggcuc........................................................................     1       1    seq
    ........................cugaagcucagagggcucu.......................................................................     1       0    seq
    ........................cugaagcucagagggcucug......................................................................     18      0    seq
    ........................cugaagcucagagggcucuga.....................................................................     76      0    seq
    ........................cugaagcucagagggcucugau....................................................................     284     0    seq
    ........................cugaagcucagagggcucugauu...................................................................     979     0    seq
    ........................cugaagcucagagggcucugauuc..................................................................     14      0    seq
    ........................cugaagcucagagggcucugauucagaaagauca........................................................     1       0    seq
    .........................ugaagcucagagggcucug......................................................................     4       0    seq
    .........................ugaagcucagagggcucuga.....................................................................     3       0    seq
    .........................ugaagcucagagggcucugau....................................................................     7       0    seq
    .........................ugaagcucagagggcucugauu...................................................................     190     0    seq
    .........................ugaagcucagagggcucugauuc..................................................................     9       0    seq
    ..........................gaagcucagagggcucugau....................................................................     3       0    seq
    ..........................gaagcucagagggcucugauu...................................................................     167     0    seq
    ..........................gaagcucagagggcucugauuc..................................................................     75      0    seq
    ..........................gaagcucagagggcucugauuca.................................................................     5       0    seq
```

oar-miR-127

```
gcuuugaucacugucuccagccugcugaagcucagagggcucugauucagaaagaucaucggauccgucugagcuuggcuggucggaagucuccucaucugcuuccuucggguu
...........................aagcucagagggcucugauu...................................................................    6      0      seq
...........................aagcucagagggcucugauuca.................................................................    3      0      seq
...........................aagcucagagggcucugauucag................................................................    6      0      seq
...........................aagcucagagggcucugauucagaa..............................................................    1      0      seq
......................................................aucaucggauccgucugagcuugg....................................    1      0      seq
.......................................................ucaucggauccgucugagcuuggcu..................................    2      0      seq
........................................................caucggauccgucugagcuugg....................................    3      0      seq
........................................................caucggauccgucugagcuuggcu..................................    1      0      seq
.........................................................aucggauccgucugagcuug.....................................    4      0      seq
.........................................................aucggauccgucugagcuugg....................................    24     0      seq
.........................................................aucggauccgucugagcuuggc...................................    46     0      seq
.........................................................aucggauccgucugagcuuggcu..................................    1      0      seq
.........................................................aucggauccgucugagcuuggcug.................................    1      0      seq
..........................................................ucggauccgucugagcu.......................................    9      0      seq
..........................................................ucggauccgucugagcuu......................................    393    0      seq
..........................................................ucggauccgucugagcuug.....................................    575    0      seq
..........................................................ucggauccgucugagcuugg....................................    9815   0      seq
..........................................................ucggauccgucugagcuuggc...................................    17324  0      seq
..........................................................ucggauccgucugagcuuggcu..................................    88653  0      seq
..........................................................ucggauccgucugagcuuggcug.................................    3674   0      seq
..........................................................ucggauccgucugagcuuggcugg................................    49     0      seq
...........................................................cggauccgucugagcuu......................................    2      0      seq
...........................................................cggauccgucugagcuug.....................................    1      0      seq
...........................................................cggauccgucugagcuugg....................................    15     0      seq
...........................................................cggauccgucugagcuuggc...................................    36     0      seq
...........................................................cggauccgucugagcuuggcu..................................    153    0      seq
...........................................................cggauccgucugagcuuggcug.................................    33     0      seq
...........................................................cggauccgucugagcuuggcugg................................    1      0      seq
............................................................ggauccgucugagcuug.....................................    1      0      seq
............................................................ggauccgucugagcuugg....................................    1      0      seq
............................................................ggauccgucugagcuuggcu..................................    6      0      seq
............................................................ggauccgucugagcuuggcug.................................    3      0      seq
```
